# Early Detection in a Mouse Model of Pancreatic Cancer by Imaging DNA Damage Response Signaling

**DOI:** 10.2967/jnumed.119.234708

**Published:** 2020-07

**Authors:** James C. Knight, Julia Baguña Torres, Robert Goldin, Michael Mosley, Gemma M. Dias, Luisa Contreras Bravo, Veerle Kersemans, P. Danny Allen, Somnath Mukherjee, Sean Smart, Bart Cornelissen

**Affiliations:** 1 Department of Oncology, CRUK/MRC Oxford Institute for Radiation Oncology, University of Oxford, Oxford, United Kingdom; 2 School of Natural and Environmental Sciences, Newcastle University, Newcastle upon Tyne, United Kingdom; and; 3 Department of Histopathology, Imperial College London, St. Mary’s Hospital Campus, London, United Kingdom

**Keywords:** pancreatic ductal adenocarcinoma, PET, SPECT, DNA damage repair, γH2AX

## Abstract

Despite its widespread use in oncology, the PET radiotracer ^18^F-FDG is ineffective for improving early detection of pancreatic ductal adenocarcinoma (PDAC). An alternative strategy for early detection of pancreatic cancer involves visualization of high-grade pancreatic intraepithelial neoplasias (PanIN-3s), generally regarded as the noninvasive precursors of PDAC. The DNA damage response is known to be hyperactivated in late-stage PanINs. Therefore, we investigated whether the SPECT imaging agent ^111^In-anti-γH2AX-TAT allows visualization of the DNA damage repair marker γH2AX in PanIN-3s in an engineered mouse model of PDAC, to facilitate early detection of PDAC. **Methods:** Genetically engineered KPC (KRas^LSL.G12D/+^; p53^LSL.R172H/+^; PdxCre) mice were imaged with ^18^F-FDG and ^111^In-anti-γH2AX-TAT. The presence of PanIN/PDAC as visualized by histologic examination was compared with autoradiography and immunofluorescence. Separately, the survival of KPC mice imaged with ^111^In-anti-γH2AX-TAT was evaluated. **Results:** In KPC mouse pancreata, γH2AX expression was increased in high-grade PanINs but not in PDAC, corroborating earlier results obtained from human pancreas sections. Uptake of ^111^In-anti-γH2AX-TAT, but not ^111^In-IgG-TAT or ^18^F-FDG, within the pancreas correlated positively with the age of KPC mice, which correlated with the number of high-grade PanINs. ^111^In-anti-γH2AX-TAT localizes preferentially in high-grade PanIN lesions but not in established PDAC. Younger, non–tumor-bearing KPC mice that show uptake of ^111^In-anti-γH2AX-TAT in the pancreas survive for a significantly shorter time than mice with physiologic ^111^In-anti-γH2AX-TAT uptake. **Conclusion:** ^111^In-anti-γH2AX-TAT imaging allows noninvasive detection of DNA damage repair signaling upregulation in preinvasive PanIN lesions and is a promising new tool to aid in the early detection and staging of pancreatic cancer.

Globally, the number of pancreatic cancer cases is predicted to reach 484,486 by 2020 (1). Approximately 90% of these patients will have pancreatic ductal adenocarcinoma (PDAC), which has a dismal 5-y survival rate of less than 5% (2). PDAC is projected to become the second most frequent cause of cancer-related death by 2030. Because current PDAC therapies are only minimally effective, the best chance of a cure is surgical resection. Most patients, however, are ineligible for surgery because they are diagnosed at an advanced stage, when the cancer has already spread beyond the pancreas. Encouragingly, if PDAC is detected when it is still confined to the pancreas, which occurs in approximately 15% of cases, the 5-y survival rate after surgery markedly increases to 25% (3) and may be improved even further by neoadjuvant chemotherapy (4). Therefore, the most effective strategy for improving PDAC survival is to detect its presence earlier so that more patients can benefit from life-extending and potentially curative surgery.

Diagnosis of pancreatic cancer usually depends on anatomic imaging techniques such as CT, endoscopic retrograde cholangiopancreatography, endoscopic ultrasound, laparoscopy, and MRI (5). This information is then used in conjunction with analysis of serum biomarkers (such as CA19-9 (6)) and biopsied tissue to confirm the diagnosis and the stage of disease (5). A major limitation of these imaging techniques is that none are sufficiently sensitive to reliably detect the molecular biomarkers of PDAC formation that arise before manifestation of anatomic abnormalities (7). In contrast, nuclear imaging techniques such as PET and SPECT do offer the required sensitivity (7). Unfortunately, however, the most widely utilized PET imaging agent, ^18^F-FDG, has been found to be ineffective for improving early detection of PDAC (7,8). Failure of this agent—which reveals abnormal glucose metabolism—is largely due to its inability to distinguish PDAC from chronic inflammation associated with focal mass-forming pancreatitis (8).

An emerging alternative strategy for early detection of PDAC involves detection of high-grade pancreatic intraepithelial neoplasias (PanIN-3) (9). Indeed, it has been suggested that the greatest hope for saving lives that otherwise would be lost to pancreatic cancer might be the early detection of PanIN precursor lesions (10). PanINs are the most common precursor of PDAC and range from PanIN-1 to PanIN-3 according to the degree of dysplasia (10).

High-grade PanIN-3 lesions have the most diagnostic value because, unlike the lower-grade lesions, these are more likely to culminate in invasive carcinoma and are generally regarded as a canonic precursor to PDAC. Unlike PanIN-1 or -2, PanIN-3 lesions are present at very low frequencies in individuals with an otherwise healthy pancreas (4%), and then only in older patients, but more so in individuals with a familial background of PDAC (11) or with pancreatitis—known risk factors for PDAC (12), whereas PanIN-3s are present in 70% of PDAC patients. Given the difficulty of assessing PanIN-3 burden noninvasively, no direct correlation with progression to PDAC can be made. However, a recent report using lineage analysis by Makohon-Moore et al. showed strong genetic relationships among high-grade PanINs and PDAC lesions from the same patient (13), confirming the model of stepwise progression from PanIN-3 to PDAC (14). Taken together, this finding suggests that detection of PanIN-3 lesions may assist in early detection of PDAC tumorigenesis.

One attractive molecular biomarker of high-grade PanIN-3 lesions is the DNA damage response (DDR) protein γH2AX, which arises through phosphorylation of the histone H2A variant H2AX and is upregulated after cellular activation of DDR signaling. The DDR machinery, including γH2AX, is well known to be elevated during the development of several cancer types (15,16), including pancreatic cancer (17). Koorstra et al. showed that γH2AX has a favorable timeline of expression as an early detection biomarker because it is highly upregulated during development of the preinvasive PanIN-3 lesions and is expressed far less in normal tissue and overt PDAC (18). Similar results were previously obtained by Bartkova et al., showing DDR activation in bladder, breast, and colon cancer precursor lesions (15).

We previously developed a SPECT imaging agent, ^111^In-anti-γH2AX-TAT (19–22), and an alternative PET imaging agent, ^89^Zr-anti-γH2AX-TAT (23), which permitted noninvasive visualization and quantification of upregulated γH2AX during tumorigenesis in a mouse model of breast cancer (20) and monitoring of response to cancer therapy in mice bearing murine PDAC allograft tumors (19). Here, employment of the ^111^In-labeled SPECT imaging agent allowed concomitant comparison by PET imaging with ^18^F-FDG. In the present study, we demonstrate the potential of this DNA damage imaging agent for the detection of high-grade PanINs during PDAC development in the genetically engineered KPC (KRas^LSL.G12D/+^; p53^LSL.R172H/+^; PdxCre) mouse model, as a proxy for the human disease.

## MATERIALS AND METHODS

Immunoconjugates were prepared and ^111^In-anti-γH2AX-TAT and ^111^In-IgG-TAT radiosynthesized using previously described methods (19), from mouse monoclonal anti-γH2AX antibody (clone JBW-301;l Merck) or isotype-matched IgG control antibody.

All animal procedures were performed in accordance with the U.K. Animals (Scientific Procedures) Act of 1986 and with local ethical committee approval. Genetically engineered KPC mice and BALB/c mice were housed in individually ventilated cages in sex-matched groups of up to 5 per cage in a facility with an artificial day–night cycle and ad libitum access to food and water.

γH2AX imaging was performed 24 h after intravenous administration of ^111^In-anti-γH2AX-TAT (5 MBq, 5 μg). ^111^In-IgG-TAT (5 MBq, 5 μg) was used as a control. In some cases, mice were also concurrently imaged using ^18^F-FDG (∼7.5 MBq), 1 h after intravenous administration. Mice were kept fasting 4 h before ^18^F-FDG injection. Groups of KPC mice were randomized for sex, whereas the wild-type mice (BALB/c) that were used as controls were all female. PET/CT and SPECT/CT images were acquired using a VECTor^4^CT scanner (MILabs). Full experimental details and reconstruction and acquisition parameters are provided in the supplemental materials (supplemental materials are available at http://jnm.snmjournals.org).

At various ages (ranging from 70 to 224 d), KPC mice underwent concomitant imaging with ^18^F-FDG and with either ^111^In-anti-γH2AX-TAT (*n* = 9) or ^111^In-anti-IgG-TAT (*n* = 8). After imaging, pancreatic tissue was harvested and processed.

To investigate the effect of pancreatic inflammation on ^111^In-anti-γH2AX-TAT uptake, BALB/c mice (*n* = 4 per group) were, in a separate study, administered cerulein via a series of 6 hourly intraperitoneal injections to induce acute pancreatitis (24). ^111^In-anti-γH2AX-TAT was administered intravenously 150 min after the last cerulein injection, and SPECT/CT imaging was performed 24 h later.

In addition, we performed a study comparing the biodistribution of ^111^In-anti-γH2AX-TAT in younger BALB/c wild-type mice (aged 66–76 d, *n* = 3) and older mice (aged 500–506 d, *n* = 3).

Separately, younger KPC mice (aged 66–77 d) without tumors (the lack of a tumor was confirmed on necropsy) were imaged by SPECT, 24 h after administration of ^111^In-anti-γH2AX-TAT (*n* = 10) or ^111^In-IgG-TAT (*n* = 8). Survival of mice was followed for up to 64 d after SPECT imaging.

To evaluate the influence of an existing tumor on the uptake of ^111^In-anti-γH2AX-TAT in KPC mice, imaging was performed 24 h after intravenous administration of ^111^In-anti-γH2AX-TAT (*n* = 9) or ^111^In-IgG-TAT (*n* = 7). The presence of tumor was confirmed on necropsy (10 mice with tumor and 6 mice without).

To determine the influence of age on the distribution of ^111^In-anti-γH2AX-TAT, 3 younger (aged 66–76 d) and 3 older (aged 500–506 d) BALB/c mice were intravenously injected with ^111^In-anti-γH2AX-TAT.

The mice were euthanized by cervical dislocation; selected organs, tissues, and blood were removed; and the percentage injected dose per gram (%ID/g) of each sample was calculated. Pancreatic tissue was flash-frozen with dry ice and stored at −80°C until required for further processing.

### Autoradiography and Histologic Analysis

Sections of pancreatic tissue were exposed to a storage phosphor screen (PerkinElmer) to generate autoradiographs. The same ex vivo tissue sections were characterized by immunofluorescence, hematoxylin and eosin, or 3,3′-diaminobenzidine staining to probe γH2AX expression and to determine PanIN/PDAC status (as defined by Hruban et al. (10)). Morphologic analysis was checked and endorsed by a qualified pathologist. Full experimental details are provided in the supplemental materials.

### Statistical Analysis

All statistical and regression analyses were performed using Prism (version 7; GraphPad Software). Linear regression with runs testing was used to check for correlations between measurements. After testing for normality using a Shapiro–Wilk test, means were compared using a *t* test with Welch correction for nonequal variances. One-way ANOVA followed by Dunnet posttesting was used to compare multiple groups. Two-way ANOVA was used to analyze grouped data. All results are reported as mean ± SD for at least 3 independent replicates, unless otherwise indicated.

## RESULTS

### γH2AX Is Upregulated During PDAC Development in KPC Mice

Using a set of pancreatic tissues obtained from KPC mice at different ages, we set out to investigate γH2AX expression during PDAC development. KPC mice exhibit invasive PDAC from 2 mo of age onward, with copresentation of precursor lesions (25). After histologic classification of tissues, we confirmed the general relationship between PanIN presentation and age in our KPC mouse colony, with older animals presenting increasing amounts of all PanIN precursor lesions (*P* < 0.0001), including high-grade PanINs (*P* = 0.0203) (Supplemental Fig. 1A). Tumor formation in our colony occurred over a large interval, with animals first showing ^18^F-FDG–avid lesions between 70 and 220 d of age. We observed no liver metastases in KPC mice in our colony.

**FIGURE 1.**
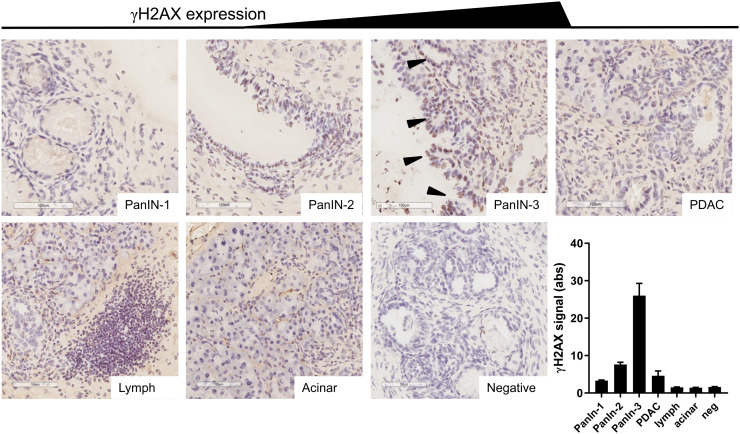
Representative examples of γH2AX staining in various types of tissue in KPC mouse pancreata. Brown indicates γH2AX, purple indicates nuclei, arrowheads indicate PanIN-3 lesions, scale bar indicates 100 μm, and bottom right panel indicates semiquantification of intensity of staining (*n* = 12). Lymph = area of focal lymphocyte infiltration.

We showed that this genetically engineered mouse model reproduces the hyperactivation of the DDR machinery observed in human PanINs and PDAC, as measured by γH2AX immunostaining (Fig. 1), first reported by Koorstra et al. (18), who themselves corroborated results from Bartkova et al., who showed DNA damage signaling hyperactivation in a large range of precursor lesions in other cancer sites (15). We observed little or no γH2AX staining in normal acinar tissue, in the earlier precursor lesions (PanIN-1), or in areas of marked lymphocyte infiltration, and we observed little γH2AX staining in PanIN-2 lesions and in regions of PDAC. However, there was marked γH2AX staining in all areas of high-grade precursor lesions (PanIN-3s). Previous reports showed very few cells expressing γH2AX foci in pancreata harvested from KC mice that only develop PanIN-1 and -2 lesions, since they lack the p53 mutation of KPC mice (26).

Since DNA laddering in the late stages of apoptosis also leads to pan-nuclear γH2AX expression, we evaluated apoptosis in sections of pancreata from KPC mice by staining for activated caspase-3 (Supplemental Fig. 2). Only in lymphocyte infiltrates, not in PDAC or PanIN lesions, was significant activated caspase-3 observed, suggesting that the γH2AX signal observed in PanIN-3 lesions is not a result of apoptosis.

### In Vivo Imaging of γH2AX Is Feasible in KPC Mice

**FIGURE 2.**
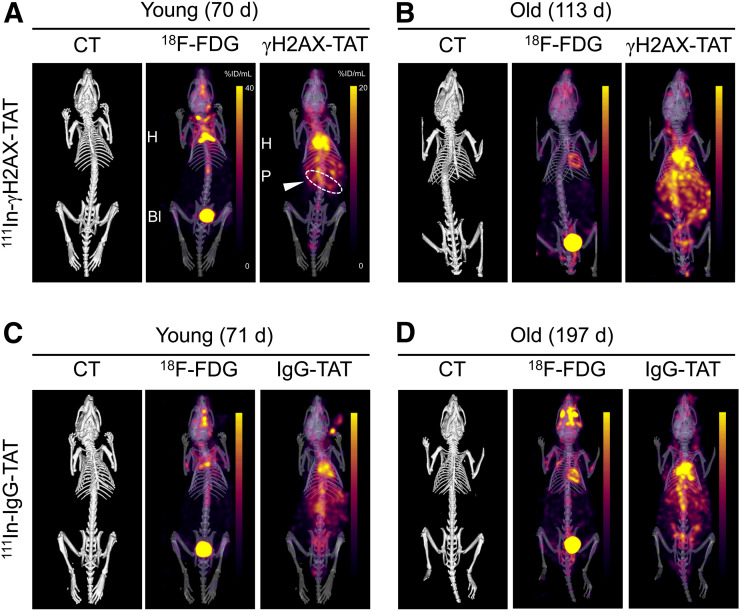
Concurrently acquired CT, ^18^F-FDG, and ^111^In-anti-γH2AX-TAT (A and B) or ^111^In-IgG-TAT (C and D) images of KPC mice. Representative examples of younger and older animals are presented. Images are coronal maximum-intensity projections superimposed on 3-dimensional rendering of CT images. Coronal images of same mice are presented in Supplemental Figure 3.

To visualize this DDR activation in KPC mice noninvasively, we performed SPECT/CT imaging 24 h after intravenous administration of ^111^In-anti-γH2AX-TAT (or the nonspecific control, ^111^In-IgG-TAT) to KPC mice of various ages. Mice that were administered ^111^In-IgG-TAT yielded SPECT images that were consistent with the typical biodistribution of a radiolabeled antibody (Fig. 2; Supplemental Fig. 3).

In contrast, some younger and all older KPC mice that were administered ^111^In-anti-γH2AX-TAT showed marked upper abdominal uptake, as well as the physiologic signal observed in blood, heart, and liver observed for ^111^In-IgG-TAT (Fig. 2). Upper abdominal uptake of ^111^In-anti-γH2AX-TAT was higher in older KPC mice. After removal of tissues from the mice, we quantified tracer uptake in a selected range of tissues (summarized in Supplemental Fig. 4). No age-related effects were seen after ^111^In-IgG-TAT administration in any of the organs, including the pancreas (*R* = 0.41, *P* > 0.05), indicating there were no nontarget-specific or clearance-related effects. In contrast, after ^111^In-anti-γH2AX-TAT administration a significant correlation was revealed between age and uptake in the pancreas (*R* = 0.83, *P* = 0.0015) (4.7 ± 0.92 vs. 6.6 ± 0.56 %ID/g in younger vs. older animals; *P* = 0.0098) but not in any other organs. This finding correlated with an increased prevalence of PanIN3 lesions, a reduced percentage of healthy pancreas, and a total increase in the relative area of PanIN lesions (Supplemental Figs. 1B–1D), as well as an increased uptake of ^111^In-anti-γH2AX-TAT, in the pancreas of tumor-bearing animals compared with non–tumor-bearing animals (where the presence or absence of a tumor was confirmed on necropsy; *P* = 0.015; Supplemental Fig. 5A). The biodistribution of ^111^In-anti-γH2AX-TAT did not significantly differ from that in age-matched wild-type BALB/c animals (aged 66–76 d; Supplemental Fig. 5B). In addition, we found no difference in organ uptake between younger and older BALB/c wild-type animals (aged 50–506 d; *P* > 0.05; Supplemental Fig. 5), suggesting that the increase in ^111^In-anti-γH2AX-TAT uptake in the pancreas was not related merely to aging of the animals.

Given the different energies of the γ-rays emitted by ^111^In (171 and 245 keV) and ^18^F (511 keV after annihilation of the positron emitted by the decaying radionuclide), we were able to image and quantify ^111^In-anti-γH2AX-TAT and ^18^F-FDG simultaneously (Fig. 2). In contrast to ^111^In-anti-γH2AX-TAT imaging, we found no significant correlation between ^18^F-FDG uptake in any of the organs and age or PanIN stage (Supplemental Fig. 4C), corroborating earlier reports on human subjects in which ^18^F-FDG PET imaging was of little utility for early detection of PDAC (7,8). Similarly, no difference in total pancreatic uptake of ^18^F-FDG was observed between BALB/c and KPC mice (*P* = 0.86; Supplemental Fig. 5B).

### γH2AX Imaging in KPC Mice Correlates with Age and PanIN Stage

**FIGURE 3.**
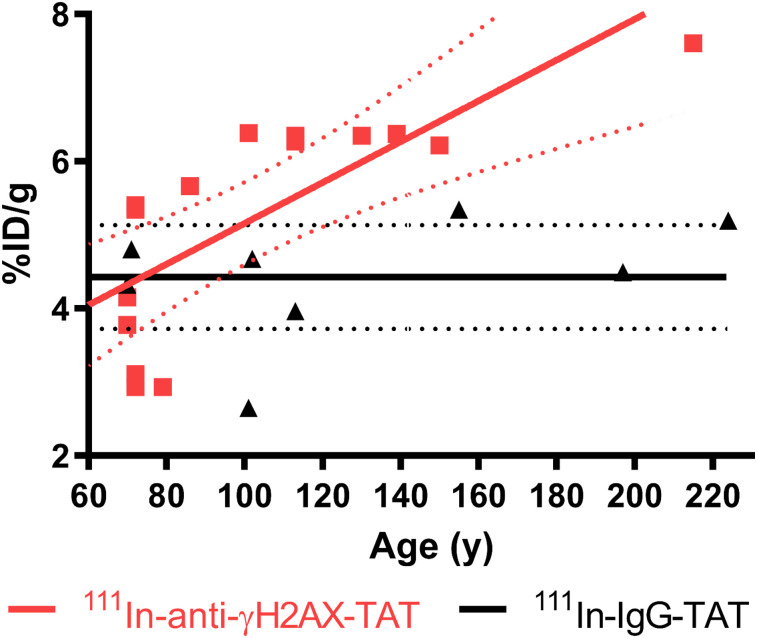
Ex vivo biodistribution data showing uptake of ^111^In in pancreas of KPC mice vs. age, after administration of either ^111^In-anti-γH2AX-TAT or ^111^In-anti-RIgG-TAT (5 MBq, 5 μg). Linear regression analysis showed no significant correlation between IgG control compound and age but did show increase in ^111^In-anti-γH2AX-TAT with age, corresponding to increased hyperplastic high-grade PanIN load in these animals.

Assessment of ^111^In-anti-γH2AX-TAT uptake in total pancreas of KPC mice, as measured by ex vivo γ-counting, revealed a clear correlation between the weight-normalized uptake of the tracer and the age of the mice (*R* = 0.83, *P* = 0.0015; Fig. 3), which in turn correlated with PanIN extent (*P* < 0.0001; Supplemental Fig. 1A). This was not the case for the nonspecific control compound ^111^In-IgG-TAT (*R* = 0.41, *P* = 0.32).

Comparing the uptake of ^111^In-anti-γH2AX-TAT in the pancreas with histologic analysis, we found a trend toward correlation with the reduction in histologically normal pancreatic tissue and increased uptake (*R* = 0.69, *P* = 0.08; Supplemental Fig. 1C). This trend was not observed for ^111^In-IgG-TAT (*R* = 0.058, *P* = 0.9). In agreement with this finding, a clear correlation was also observed for the coverage of total PanIN lesions and pancreatic uptake of ^111^In-anti-γH2AX-TAT (*R* = 0.73, *P* = 0.031) but not with ^111^In-IgG-TAT (*R* = 0.0015, *P* = 0.99) (Supplemental Fig. 1D).

### ^111^In-Anti-γH2AX-TAT Is Taken Up Only in High-Grade PanIN Lesions

**FIGURE 4.**
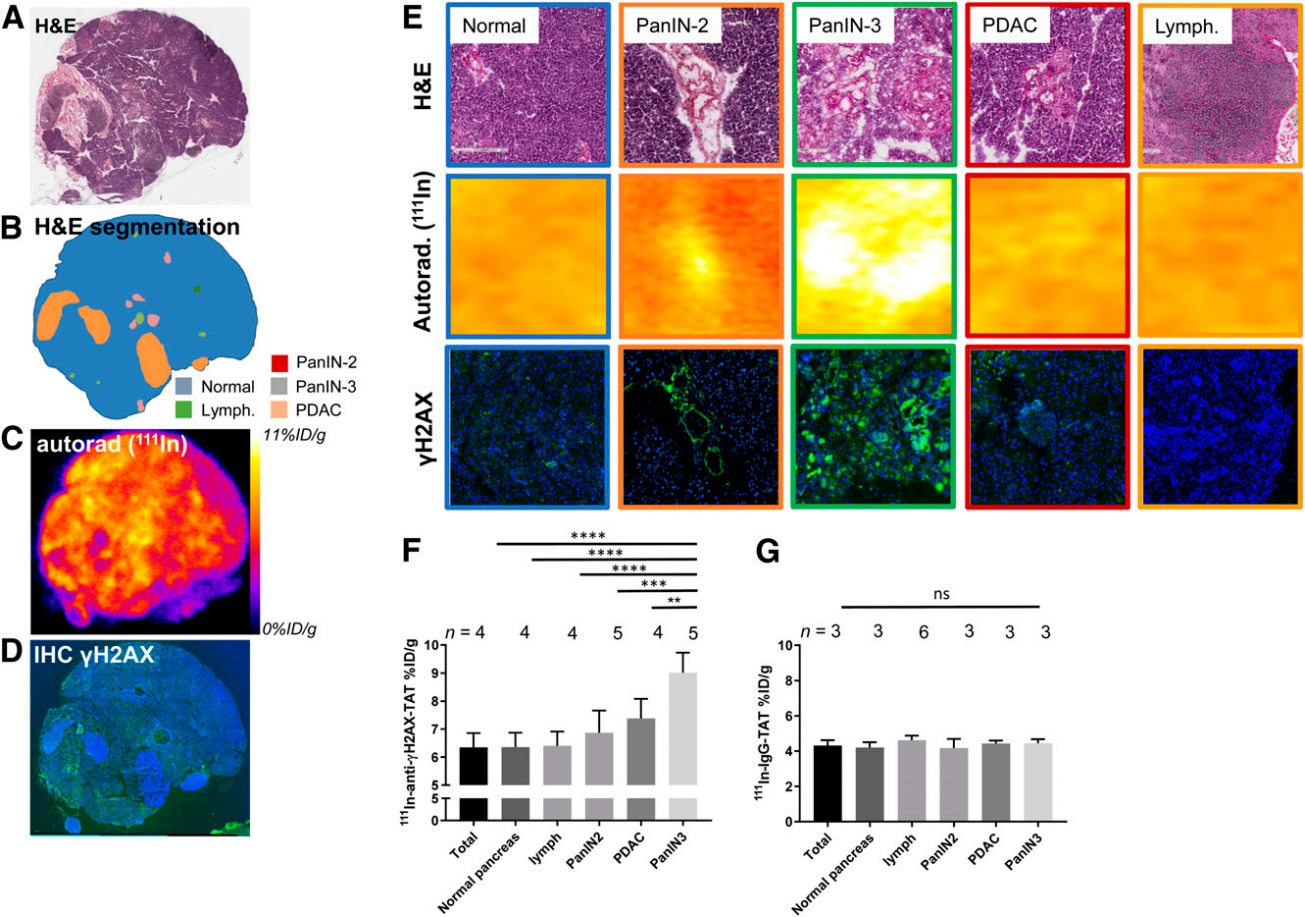
(A) Hematoxylin and eosin staining of pancreas section from 113-d-old KPC mouse harvested 24 h after administration of ^111^In-anti-γH2AX-TAT. (B) Identification of various morphopathologic features. (C) Autoradiography image showing distribution of radioactivity. (D) Immunofluorescence image showing γH2AX (green) and nuclei (blue). Additional sections are shown in Supplemental Figure 6. (E) Magnification of histologic areas in A–D. (F) Uptake of ^111^In-anti-γH2AX-TAT in various morphopathologic features in KPC pancreata, measured by ex vivo autoradiography of pancreas sections. (G) Uptake of ^111^In-IgG-TAT in various morphopathologic features in KPC pancreata, measured by ex vivo autoradiography of pancreas sections. Autorad = autoradiography; H&E = hematoxylin and eosin; IHC = immunohistochemistry; lymph = lymphocytes.

To confirm that systemically delivered ^111^In-anti-γH2AX-TAT colocalizes with high-grade PanIN lesions in vivo, we performed autoradiography and immunohistochemistry probing for γH2AX on sections of pancreatic tissue obtained from KPC mice (Fig. 4; additional sections are shown in Supplemental Fig. 6). In line with our earlier results that γH2AX expression was observed mostly in high-grade PanIN-3 tissue, a significantly higher autoradiography signal after administration of ^111^In-anti-γH2AX-TAT was observed in high-grade PanIN-3s than in normal pancreas, early PanIN lesions, lymphocyte infiltrates, or PDAC, although some background uptake was observed in normal pancreatic tissue. Quantitative analysis of autoradiographs after ^111^In-anti-γH2AX-TAT imaging corroborated this observation, with high-grade PanIN lesions showing an uptake of 9.01 ± 0.72 %ID/g, versus 6.35 ± 0.51 %ID/g for the total pancreas (*P* < 0.0001), lymphocyte infiltrates (*P* = 0.0001), PanIN2 lesions (*P* = 0.003), or PDAC tissue (*P* = 0.0073) (Fig. 4F). No such correlation was observed in mice that were administered ^111^In-IgG-TAT (*P* > 0.05; Fig. 4G). Although we observed a small increase in γH2AX staining in PanIN2 lesions on immunohistochemical analysis, this increase did not result in a significant increase in uptake of ^111^In-anti-γH2AX-TAT in these lesions, as measured by autoradiography (*P* > 0.05).

In addition, we observed no increased uptake of ^111^In-anti-γH2AX-TAT in lymphocyte infiltrates, compared with normal pancreas (*P* = 0.0001). Furthermore, in a separate study, no increased uptake of ^111^In-anti-γH2AX-TAT was measured in the pancreas of mice with cerulein-induced acute pancreatitis (*P* > 0.05; Supplemental Fig. 7).

### ^111^In-Anti-γH2AX-TAT Uptake Predicts Onset of PDAC in KPC Mice

**FIGURE 5.**
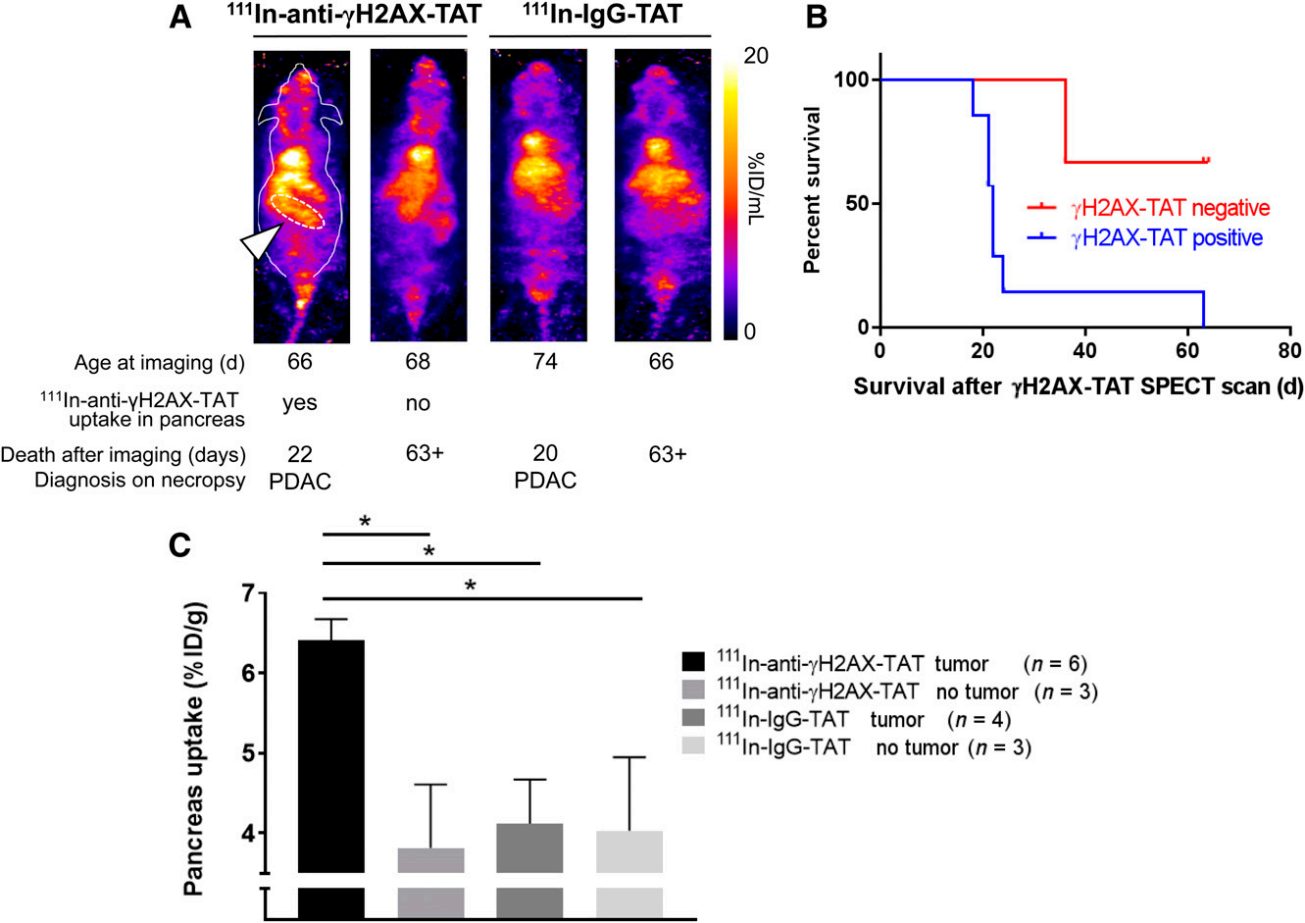
(A) Representative images of KPC mice aged 66–77 d imaged by SPECT, 24 h after intravenous administration of ^111^In-anti-γH2AX-TAT. Age at time of imaging, length of survival before clinical symptom endpoints were reached, and diagnosis at necropsy are indicated for each mouse. Pancreatic region is indicated by white arrowhead and white dashed line in first animal only. Coronal maximum-intensity projections are shown; outline of mouse is indicated for first animal only. (B) Mice showing uptake of ^111^In-anti-γH2AX-TAT in pancreas had significantly shorter survival than those not showing pancreatic uptake (*P* = 0.0273). (C) ^111^In-anti-γH2AX-TAT, but not ^111^In-IgG-TAT, was taken up more in pancreata of tumor-bearing KPC mice.

After showing that ^111^In-anti-γH2AX-TAT is taken up preferentially in high-grade PanINs (i.e., PanIN-3s), and given that these lesions are regarded as direct precursors of PDAC, we evaluated, in a separate study, the ability of SPECT imaging with ^111^In-anti-γH2AX-TAT to predict tumor formation and survival. KPC mice aged between 66 and 77 d were imaged by SPECT, 24 h after administration of ^111^In-anti-γH2AX-TAT (*n* = 11) or control imaging agent ^111^In-IgG-TAT (*n* = 8). Mice with increased uptake of ^111^In-anti-γH2AX-TAT in the pancreas region (as indicated in Fig. 5A) survived a significantly shorter time than those with little discernible uptake (median survival, 22 d vs. more than 63 d, respectively; *P* = 0.0273). No such observation was made for mice imaged with ^111^In-IgG-TAT (all scans showed little to no uptake in the pancreas region, similar to Fig. 2C). All images obtained are shown in Supplemental Fig. 7. Mice were randomly assigned to ^111^In-anti-γH2AX-TAT or ^111^In-IgG-TAT imaging cohorts, which had no influence on survival (*P* > 0.05; Supplemental Fig. 8). In addition, no correlation was found between the age at which these animals were imaged and their survival after the scan (*P* > 0.05; Supplemental Fig. 8). Together with the observation that no increased uptake of ^111^In-anti-γH2AX-TAT is detected in age-matched wild-type mice (Supplemental Fig. 5B), our results suggest that visualizing the high-grade PanIN-3 lesions using ^111^In-anti-γH2AX-TAT can be used as an indicator of PDAC development in KPC mice. A caveat here is that of course all KPC mice will eventually grow tumors. The true specificity and selectivity of the method can therefore not be determined using the experimental setup in this proof-of-principle study.

In a separate study, we evaluated the effect of the presence, or not, of a tumor (confirmed on necropsy) on the uptake of ^111^In-anti-γH2AX-TAT or the control compound ^111^In-IgG-TAT. We observed increased uptake of ^111^In-anti-γH2AX-TAT, but not of ^111^In-IgG-TAT, in the whole pancreas in KPC mice bearing a tumor, compared with non–tumor-bearing animals (*P* < 0.05; Fig. 5C), consistent with a higher PanIN-3 burden in resected pancreata of PDAC patients. None of the other organs showed significant differences in uptake (*P* > 0.05; Supplemental Fig. 5B), apart from some inconsistent variation in kidney and spleen values.

## DISCUSSION

Despite recent progress, there remains an urgent need for imaging methodologies for early detection of PDAC (27,28). A decade ago, Koorstra et al. described a hyperactivation of the DDR machinery during PDAC development, with increasing expression of activated ATM (p(Ser1981)ATM) and its substrates pCHK2 and γH2AX in high-grade hyperplasias (PanIN-3 lesions) compared with normal tissue and low-grade PanINs, but less so in PDAC (18). This study was consistent with earlier data from Bartkova et al., who showed similar findings in a wide variety of other tumor types (15). Here, we confirmed that the KPC mouse model of PDAC replicates this biology, with a marked increase in γH2AX staining in PanIN-3 lesions. As in the human disease, DDR hyperactivation in KPC mice subsides in lesions reaching the PDAC stage. This characteristic offers the possibility of specifically visualizing PanIN-3 precursor lesions to complement currently used imaging technologies, such as ultrasound, CT, and MRI, that can detect only the later-stage, larger invasive PDAC lesions.

We demonstrate here that γH2AX imaging using ^111^In-labeled, TAT-modified anti-γH2AX IgG can be used to detect high-grade PanINs in vivo, because of the propensity of these lesions to express higher levels of the phosphorylated γH2AX protein. Earlier studies had already shown that the ubiquitously used PET imaging agent ^18^F-FDG does not clearly delineate these precursor lesions (24). Although the differences in uptake of ^111^In-anti-γH2AX-TAT described for PanIN3 versus other tissues are subtle, a clear benefit has been shown in survival studies. Our results here in a pancreatic cancer model confirm our earlier findings obtained in BALB/neuT mice, a genetically engineered mouse model of HER2-driven breast cancer (20). Notably, as before, no toxicity was observed from targeting an antibody to a DDR protein (23). Modeling showed previously that ^111^In-anti-γH2AX-TAT caused no significant difference in DNA double-strand break (DSB) repair or in clonogenic survival (29). On the other hand, we previously showed that, when increasing amounts of ^111^In were attached to anti-γH2AX-TAT (≤6 MB/μg), the Auger electron emissions from ^111^In could result in formation of additional DNA DSB damage and, consequently, more γH2AX foci, resulting in an autoamplification loop and increased cell kill. A similar scenario may be proposed in which the DDR hyperactivation in pretumorous lesions is targeted by this therapeutic version of anti-γH2AX-TAT.

We observed marked increases in total pancreatic uptake of ^111^In-anti-γH2AX-TAT, even when only a small portion of the pancreas was classed as diseased PanIN-3. In addition, we measured some signal in nondiseased pancreatic tissue in KPC mice or in pancreas in naïve wild-type animals. These findings suggest that uptake in the small PanIN-3 lesions is enough to enable macroscopic detection using the imaging agent. Importantly, it has been observed that, on careful resection of whole pancreata, multiple advanced PanIN-3 lesions are the norm rather than the exception and that PanIN is a disease that is able to spread through the entire ductal system (12,13), with densities of up to 0.16 lesions histologically detectable per square centimeter (30). This characteristic may aid detection in the clinical setting, in which they may be used in conjunction with CT and MRI-based methods already in use for screening of high-risk patients or guide more invasive sampling. The technology may also be used to further study the temporal relationship between PanIN3 burden and PDAC formation. In addition, detection of PanIN-3 burden may act as a prompt or a therapy efficacy readout for PanIN-3 ablation therapies.

Some of the limitations of our approach derive from use of a whole antibody to target γH2AX. Not only does this makes the agent more difficult to produce than small-molecule or peptide-based imaging vectors, but antibodies are generally slow to clear from blood circulation and are taken up by the liver and spleen (7), potentially limiting the ability of any antibody-based imaging agent to detect liver metastases. Nonetheless, the modified antibody we used here does afford an excellent selectivity and affinity (dissociation constant, 23 nM) for its target epitope. Meanwhile, no other imaging vectors have yet been identified that reliably detect γH2AX or any other posttranslational modifications of the phosphorylated serines and threonines that make up the bulk of information passed on in the DDR signaling networks.

Despite being well characterized, γH2AX expression is used mostly in the context of DNA DSBs. However, γH2AX expression is not exclusively linked to DSBs, as γH2AX is also expressed around collapsed replication forks, and γH2AX expression can persist for hours or even days after any DSBs have long since been repaired (21). Although these considerations do raise a cautionary note for the interpretation of γH2AX imaging in general, they do not take away from its ability to act as a biomarker for pretumorous lesions, including PanIN-3s. It is also noteworthy that γH2AX expression is observed in the late stages of apoptosis but that we observed no correlation between apoptotic cells, as highlighted by activated caspase-3, and γH2AX staining or ^111^In-anti-γH2AX-TAT uptake, perhaps aided by the short-lived nature of late apoptotic cells, causing only little contribution to the macroscopic signal observed by autoradiography or SPECT imaging. In addition, we observed no γH2AX-positive micronuclei resulting from genomic instability, which may confound the detection of DDR upregulation by noninvasive imaging (31). Finally, multiple links have been shown to exist between DNA damage signaling and inflammatory signaling, and DDR signaling has been shown to influence immune surveillance of tumor tissue (32). Moreover, pancreatic inflammation is a key risk factor for pancreatic cancer (33), and this inflammation can directly affect epithelial cells to generate reactive oxygen and nitrogen species that lead to DNA damage and result in genetic instability. However, we observed no γH2AX staining in lymphocyte infiltrates in KPC mouse pancreata, suggesting that—at least in this setting—imaging of PanIN-3s with ^111^In-anti-γH2AX-TAT may be independent of the presence of immune cells. In addition, we observed no increased uptake in mice with cerulein-induced acute pancreatitis.

We have previously demonstrated that γH2AX imaging in PDAC can be used to detect DSB induction by cancer drugs or radiation (34). In clinical practice, this use would enable early detection of treatment response, thereby allowing an early switch to alternative therapies in nonresponding patients. We now demonstrate that γH2AX imaging may allow early detection of precancerous PanIN-3 precursor lesions. The data we present in this article were obtained using a mouse model of PDAC only. The staining quantification we used here was distinct from the histoscores used by Koorstra et al. (18). Although it capably mimics clinical tumorigenesis of PanINs to PDAC, with similar γH2AX expression patterns, PET imaging with ^89^Zr-anti-γH2AX-TAT in PDAC patients and correlation between ex vivo autoradiography and PanIN histopathology are the next necessary steps to validate this technology for human use.

## CONCLUSION

In a preclinical genetically engineered mouse model of PDAC, we have shown for the first time, to our knowledge, the possibility of imaging the hyperactivation of DNA damage repair signaling during pancreatic tumorigenesis, a direct result of genomic stress caused by oncogenic transformation. This novel tool may be used as a part of focused surveillance in individuals at increased risk of developing PDAC.

## DISCLOSURE

This research was supported by CRUK through the Oxford Institute for Radiation Oncology and the CRUK Oxford Centre and by the CRUK/EPSRC Imaging Centre in Oxford and Pancreatic Cancer UK. Further support was obtained from Pancreatic Cancer U.K. (Bart Cornelissen and Luisa Contreras Bravo) and the Pancreatic Cancer Research Fund (Bart Cornelissen and Julia Baguña Torres). Julia Baguña Torres is funded through a project grant from the Pancreatic Cancer Research Fund. No other potential conflict of interest relevant to this article was reported.

**KEY POINTS**

**QUESTION:** Can DNA damage hyperactivation in preinvasive cancer-precursor lesions act as a means of early detection of pancreatic cancer by molecular imaging?
**PERTINENT FINDINGS:** In this study, we found that hyperactivation of DNA damage repair signaling in PanIN-3 lesions in a genetically engineered mouse model of spontaneous PDAC could be highlighted by molecular imaging targeting γH2AX. This finding permitted prediction, at an early stage, of which mice would develop PDAC.
**IMPLICATIONS FOR PATIENT CARE:** The method described in this paper may allow early detection of cancer in high-risk patient groups.
