# Supplementary material for: Early Detection in a Mouse Model of Pancreatic Cancer by Imaging DNA Damage Response Signaling
Source: J Nucl Med. 2020 Jul;61(7):1006–13. doi: 10.2967/jnumed.119.234708 (PMC7383084; doi:10.2967/jnumed.119.234708)
Supplement: Supplementary file 1 [file jnm234708SupplementalData.pdf]

PET/SPECT/CT Imaging

General

PET/SPECT/CT imaging was performed using a VECTor[4] imaging system (MILabs) using a rat collimator with 1.8 mm pinholes. List-mode PET/SPECT data were acquired for 30 minutes and a CT image was then subsequently acquired (55 kV, 0.19 mA) on the same system. For the indium-111 SPECT images, reconstructions were performed using a γ-ray energy window of 156–190 keV (background weight 2.5), 0.6 mm^3 voxels, 128 subsets, and 2 iterations using the manufacturer's POSEM reconstruction protocol. For the fluorine-18 PET images, reconstructions were performed using energy windows of 460-562 keV (background weight 2.5), 0.8 mm^3 voxels, 128 subsets, and 5 iterations using the manufacturer's POSEM reconstruction type. To allow accurate scaling and quantification, calibration factors (determined from phantoms) were applied to the data. PET and SPECT images were each registered to CT and then attenuation corrected. During each imaging session, mice were kept under anaesthesia by inhalation of 2% isofluorane in air and maintained at 37°C. Images were processed and volume-of-interest analyses were performed using the PMod software package (version 3.807, PMOD Technologies).

^{111}In-anti-γH2AX-TAT and ^{18}F-FDG imaging in ageing KPC mice

At various ages (ranging from 70 to 224 days), KPC mice underwent dual-isotope (^{18}F and ^{111}In) PET/SPECT imaging at 1 h after administration of ^{18}F-FDG, and at 24 h after injection of either ^{111}In-anti-γH2AX-TAT (n = 9) or ^{111}In-anti-IgG-TAT (n = 8). After imaging, selected tissues and blood were harvested, and the percentage of the injected dose of radiolabelled compound per gram in each of them was determined. Pancreatic tissue was snap-frozen, cyrosectioned, and processed for autoradiography, γH2AX immunohistochemistry and haematoxylin and eosin (H&E) staining.

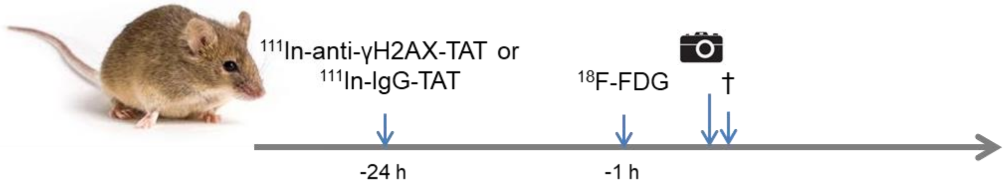

^{111}In-anti-γH2AX-TAT imaging and survival in KPC mice

KPC mice (aged 66-77 days) without tumours were imaged by SPECT, 24 h after administration of ^{111}In-anti-γH2AX-TAT (n = 10) or ^{111}In-IgG-TAT (n = 8) as above. Survival of mice was followed for up to 64 days after SPECT imaging. Mice were euthanised when they showed the first clinical symptoms of disease. Diagnosis of PDAC was confirmed on necropsy.

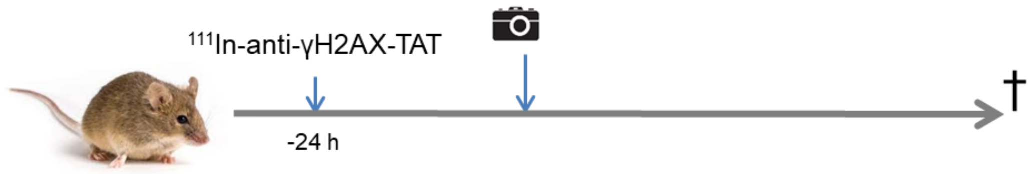

^{111}In-anti-γH2AX-TAT imaging in tumour-bearing KPC mice

To evaluate the influence of an existing tumour on the uptake ^{111}In-anti-γH2AX-TAT in KPC mice, animals were euthanised 24 h after intravenous administration of ^{111}In-anti-γH2AX-TAT (n = 9) or ^{111}In-IgG-TAT (n = 7). Selected organs and tissues were harvested and the percentage of the injected dose per gram of tissue was determined. Pancreata were measured with the tumour(s) if present. Tumour presence was confirmed on necropsy (10 mice with tumour and 6 mice without tumour were included in this study).

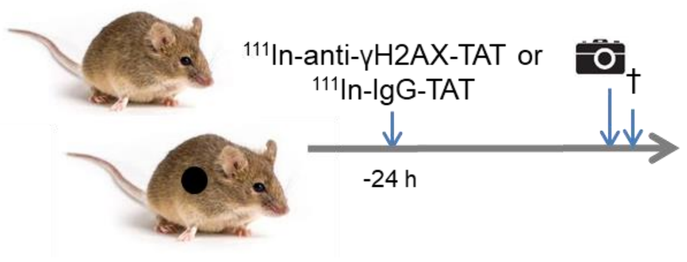

^{111}In-anti-γH2AX-TAT imaging in older wild type mice

To determine the influence of age on the distribution of ^{111}In-anti-γH2AX-TAT, three younger (aged 66-76 days) and three older (aged 500-506 days) mice were intravenously injected with ^{111}In-anti-γH2AX-TAT. 24 h later, selected organs and blood were harvested and the percentage of the injected dose per gram of tissue was determined.

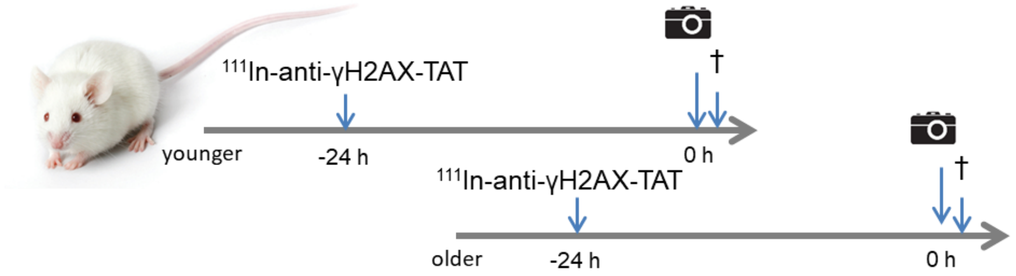

^{111}In-anti-γH2AX-TAT imaging in mice with acute pancreatitis

Caerulein, sulfated (MPBIO, UK) was dissolved in 0.9 % saline for an injectable dose of 50 ug/kg in 30 µL. C57BL/6 female mice, 10 weeks of age were injected (IP) with either caerulein or saline over a period of 6 hours. At 7.5 hours post the initial caerulein or saline injection all mice were injected via the tail vein with ^{111}In-DTPA-γH2Ax-TAT (4.5 MBq, 5ug).

SPECT/CT images were acquired 24 h later, for approximately 30 min using a VECTor^{4}CT scanner (MILabs, Utrecht, the Netherlands) as above. Immediately after imaging, mice were euthanised by cervical dislocation and selected organs, tissues and blood were removed. The samples were immediately rinsed with water, dried, and transferred into a pre-weighed counting tube. After weighing the filled counting tubes, the amount of radioactivity in each was measured using a HiDex gamma counter (Lablogic). Counts per minute were converted into radioactivity units (MBq) using calibration curves generated from known standards. These values were decay-corrected to the time of injection, and the percentage of the injected dose per gram (%ID/g) of each sample was calculated. Pancreatic tissue was flash-frozen with dry ice and stored at -80°C until required for further processing.

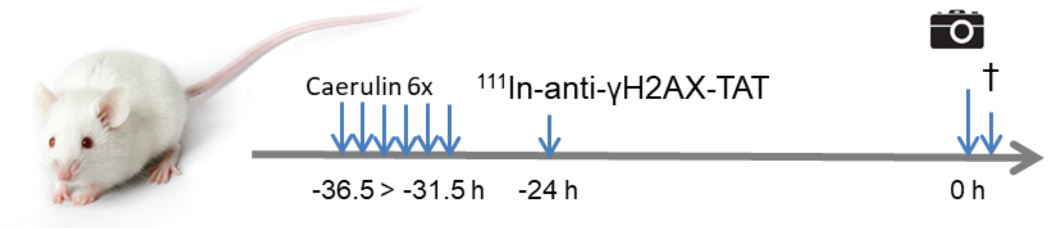

Ex vivo biodistribution experiments

After imaging (unless long-term follow-up was necessary), mice were euthanised by cervical dislocation and selected organs, tissues and blood were removed. The samples were immediately rinsed with water, dried, and transferred into a pre-weighed counting tube. After weighing the filled counting tubes, the amount of radioactivity in each was measured using a 2480 WIZARD^2 (PerkinElmer) or HiDex gamma counter (Lablogic). Counts per minute were converted into radioactivity units (MBq) using calibration curves generated from known standards. These values were decay-corrected to the time of injection, and the percentage of the injected dose per gram (%ID/g) of each sample was calculated. Pancreatic tissue was flash-frozen with dry ice and stored at -80°C until required for further processing.

Immunofluorescence Imaging

Pancreases harvested from mice (n = 12) were flash frozen and 8 μm sections were prepared using a cryostat. Sections were stored at -80°C until use. Slides were allowed to reach room temperature for 10 minutes then washed briefly in phosphate-buffered saline (PBS) pH 7.4. The slides were fixed in 4% paraformaldehyde/PBS for 10 min, then washed three times in PBS for 5 min. Sections were permeabilized in 1% Triton X-100 for 10 min, washed, and non-specific binding was blocked by incubation of the slides in 2% BSA/PBS + 0.1% Triton X-100 for 1 h at 37°C. Slides were briefly allowed to dry and each section was isolated using a PAP pen. To each appropriate section approximately 100 µL of primary anti-γH2AX monoclonal antibody (Merck, catalogue no. 05-636, clone JBW301) diluted 1:250 in 2% BSA/PBS + 0.1% Triton X-100, or just 2% BSA/PBS + 0.1% Triton X-100, was applied, and incubated for 1 h at 37°C. The slides were washed three times in PBS for 5 min and the secondary goat anti-mouse Alexa Fluor-488 antibody (ThermoFisher Scientific, catalogue no. A-11001) diluted 1:250 in 2% BSA/PBS was applied and incubated for 1 h at 37°C. The slides were then washed in PBS, excess fluid removed, mounted using Vectashield containing DAPI (Vector Laboratories, catalogue no. H-1200). Slides were stored at 4°C in the dark. Images were acquired using a Leica SP8 confocal fluorescent microscope.

Autoradiography

Sections of pancreas tissue were exposed to a storage phosphor screen (PerkinElmer) to generate autoradiographs. Quantification was performed in one section each from at least three animals (as indicated) by ImageJ, using regions-of-interest based on H&E-based morpho-pathological features. The average intensity per VOI was used as a metric, and converted to %ID/g using known standards.

3,3'-Diaminobenzidine (DAB) and Haematoxylin Staining

To prevent staining of endogenous peroxidases, pancreas sections were incubated with 1.5% H_2O_2 for 15 minutes at room temperature and then rinsed with deionised water. Slides were briefly allowed to dry and each section was isolated using a PAP pen. Non-specific binding was blocked by incubation of the slides in 5% goat serum in PBS + 0.1% Tween-20 for 1 h at 37°C. To each section approximately 100 µL of primary anti-γH2AX monoclonal antibody (Merck, catalogue no. 05-636, clone JBW301) diluted 1:800 in 5% goat serum in PBS + 0.1% Tween-20, or just 5% goat serum in PBS + 0.1% Tween-20, was applied, and incubated for 1h at room temperature. The slides were washed three times in PBS for 5 min and the secondary goat anti-mouse antibody (BA-9200, Vector Laboratories) diluted 1:250 in 5% goat serum in PBS + 0.1% Tween-20 was applied and incubated for 30 minutes at room temperature. The slides were then washed three times in PBS + 0.1% Tween-20 for 5 minutes. ABC-HRP (Vactastain Elite, ABC-HRP reagent, R.T.U. peroxidase, PK-7100) was then applied to each section and incubated for 30 minutes at room temperature. The slides were then washed three times in PBS + 0.1% Tween-20 for 5 minutes. DAB solution (Pierce, DAB substrate kit) was then applied to each section and incubated for 5 minutes. The slides were then washed three times with deionised water for 5 min and then counterstained with haematoxylin for 2 minutes. Slides were washed in running tap water and then dehydrated with an increasing concentration gradient of ethanol. Slides were mounted using DPX mounting medium and then stored at 4°C in the dark. Images were acquired at a 20x magnification using an Aperio Slide Scanner (Leica Biosystems) and analysed using the Aperio ImageScope Viewer (Leica Biosystems).

Supplemental Fig. 1

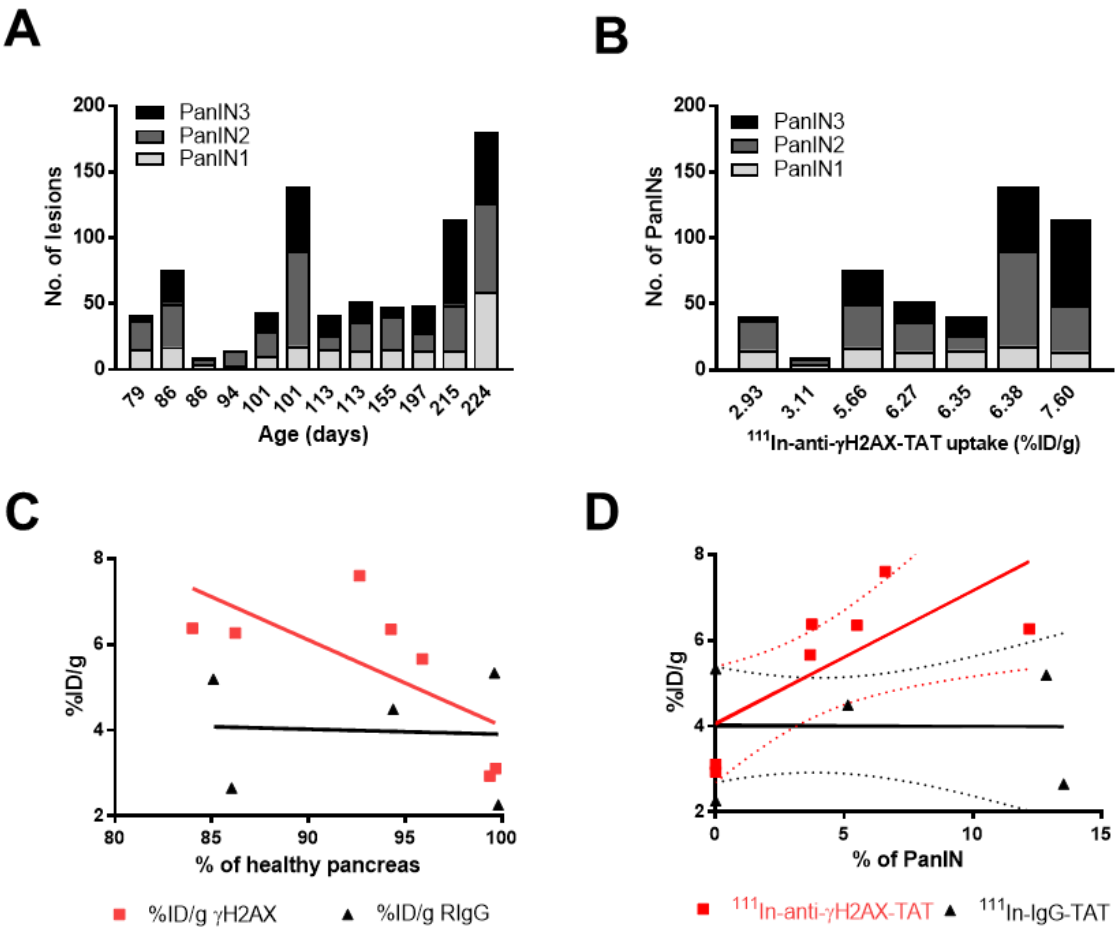

Supplemental Fig. 1 (A) Quantification of the extent of PanIN1, 2, and 3 involvement in KPC mice of varying ages shows a clear correlation of PanIN3 presentation with age (n = 12). (B) Consistent with higher γH2AX expression in PanIN3 lesions, uptake of ^{111}In-γH2AX-TAT in pancreata of KPC mice correlates with the amount of PanIN3 lesions (n = 7). (C) Similarly, uptake of ^{111}In-anti-γH2AX-TAT, but not ^{111}In-IgG-TAT, in KPC mouse pancreas decreases with an increasing amount of healthy pancreatic tissue (n = 7 and 5, respectively). (D) Corroborating these observations, there is a trend towards a correlation between the extent of PanIn3 lesions and ^{111}In-γH2AX-TAT uptake in KPC mouse pancreas (n = 7 and 5, respectively).

Supplemental Fig. 2

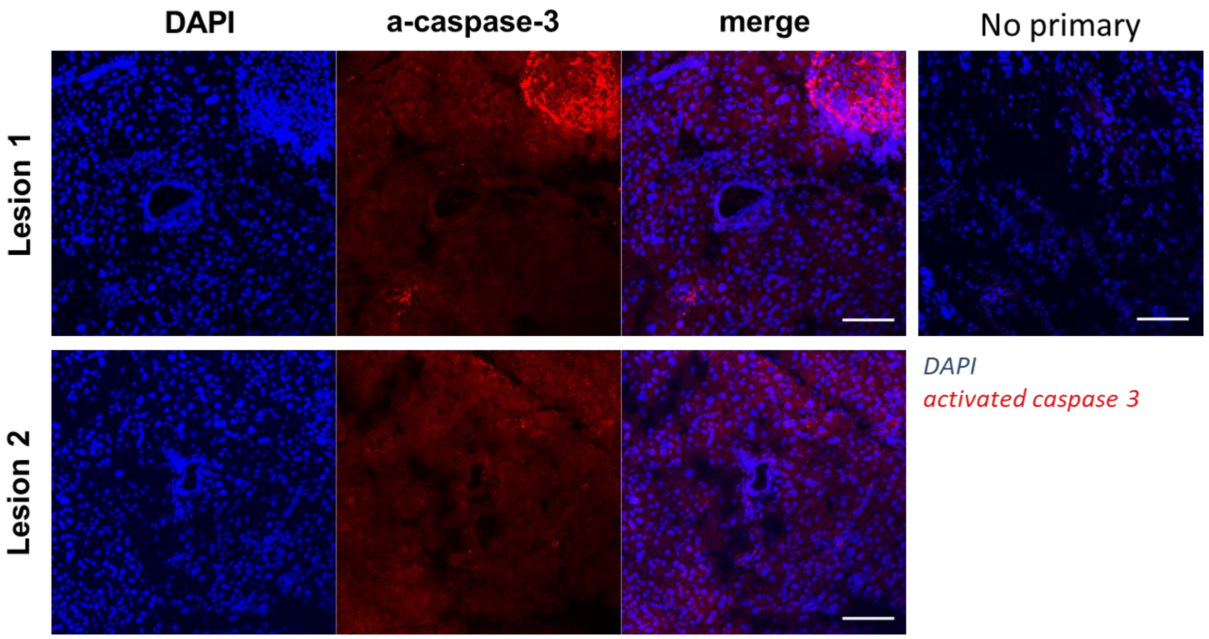

Supplemental Fig. 2 Activated caspase-3 staining in KPC mouse pancreas sections reveals little to no caspase activation in two independent PanIN3 lesions. No correlation with tissue positive for γH2AX were observed. Scale bar indicates 50 μm. A negative control where the primary anti-capsase-3 antibody was omitted is also included to demonstrate the lack of non-specific staining or autofluorescence.

Supplemental Fig. 3

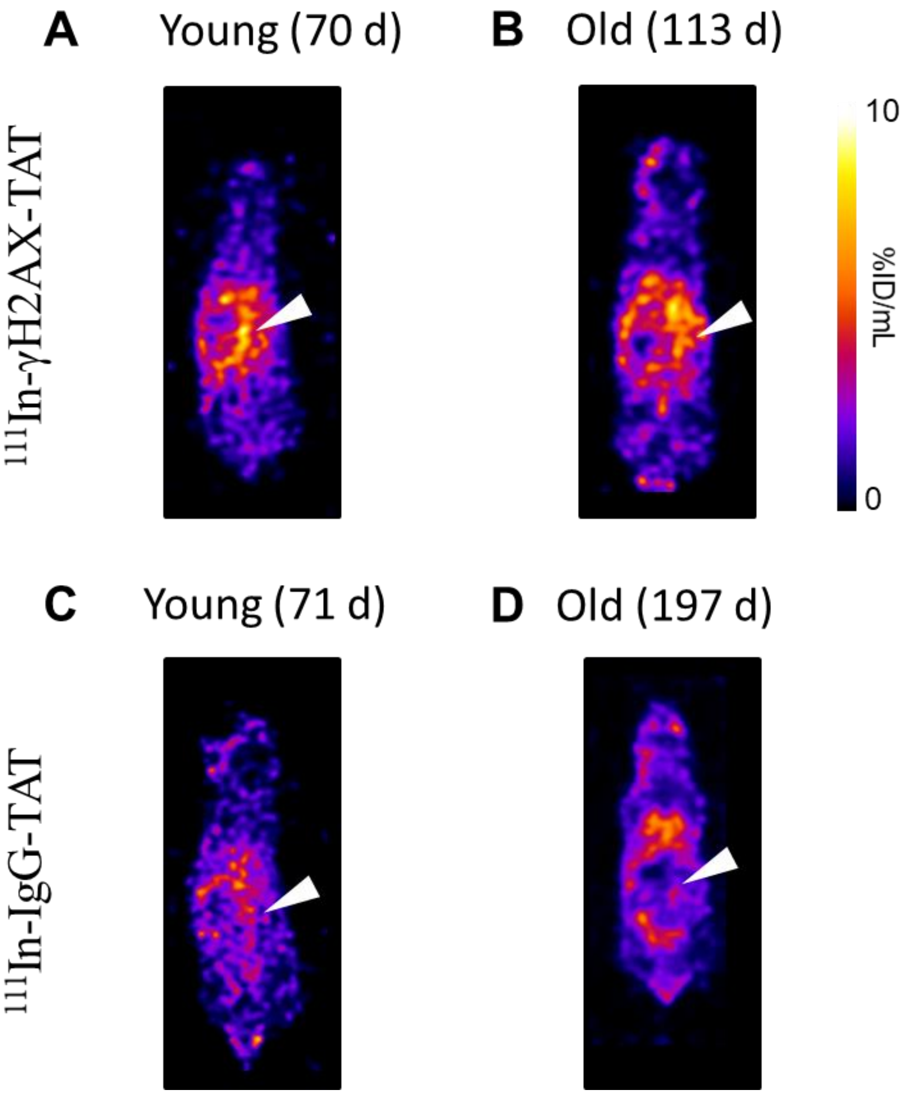

Supplemental Fig. 3: ^{111}In-anti-γH2AX-TAT (A, B) or ^{111}In-IgG-TAT (C, D) imaging in KPC mice. Representative examples of either younger (A, C) or older animals (B, D) are presented. Image are shown as coronal sections through the pancreas (indicated by the white arrowhead).

Supplemental Fig. 4

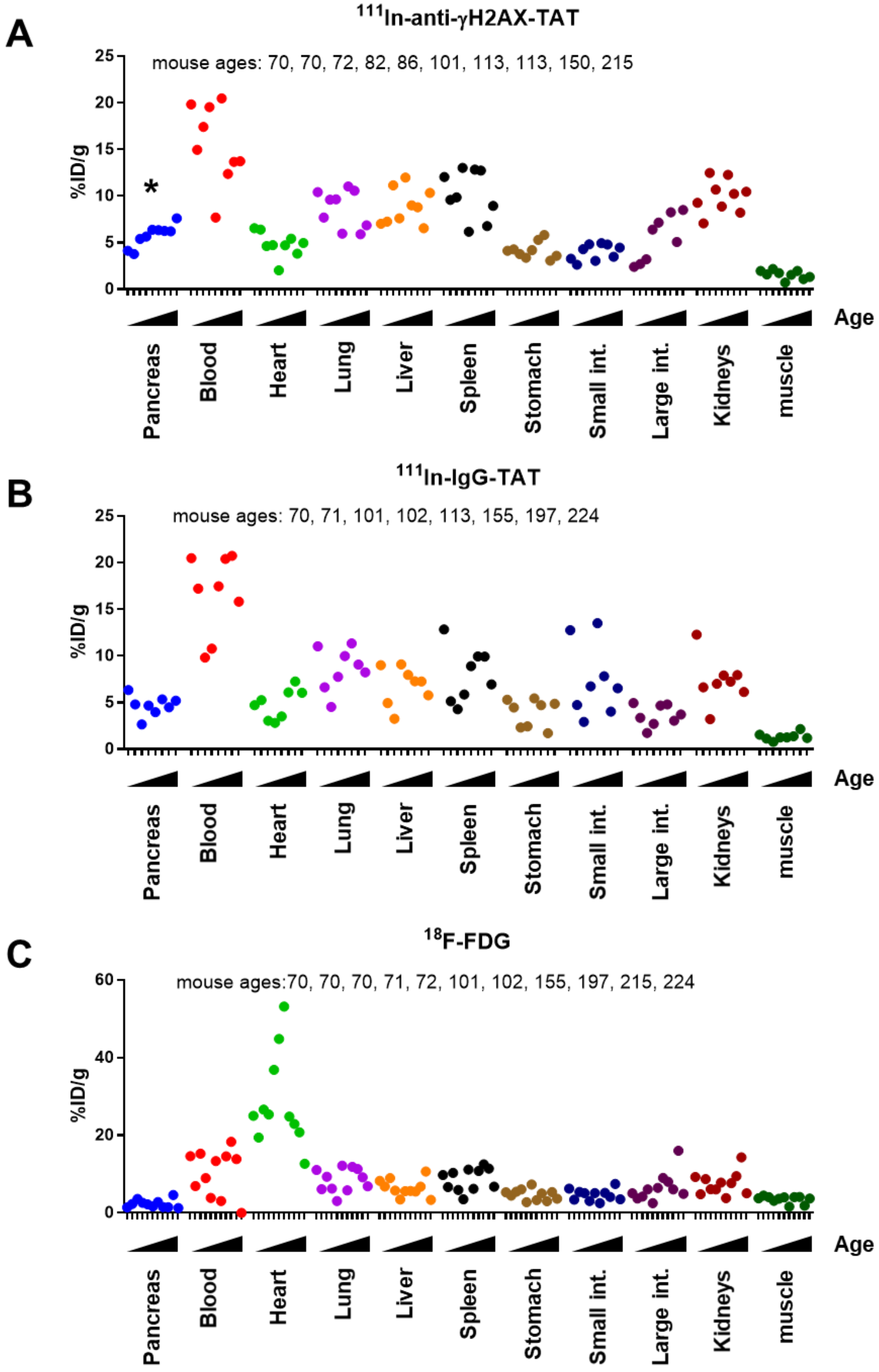

Supplemental Fig. 4 *Biodistribution of ^{111}In-anti-γH2AX-TAT (A), ^{111}In-IgG-TAT (B), or ^{18}F-FDG (C) in KPC animals of various ages. Results are expressed as the percentage of the injected dose per gram of tissue. (* significant correlation of %ID/g with age)*

Supplemental Fig. 5

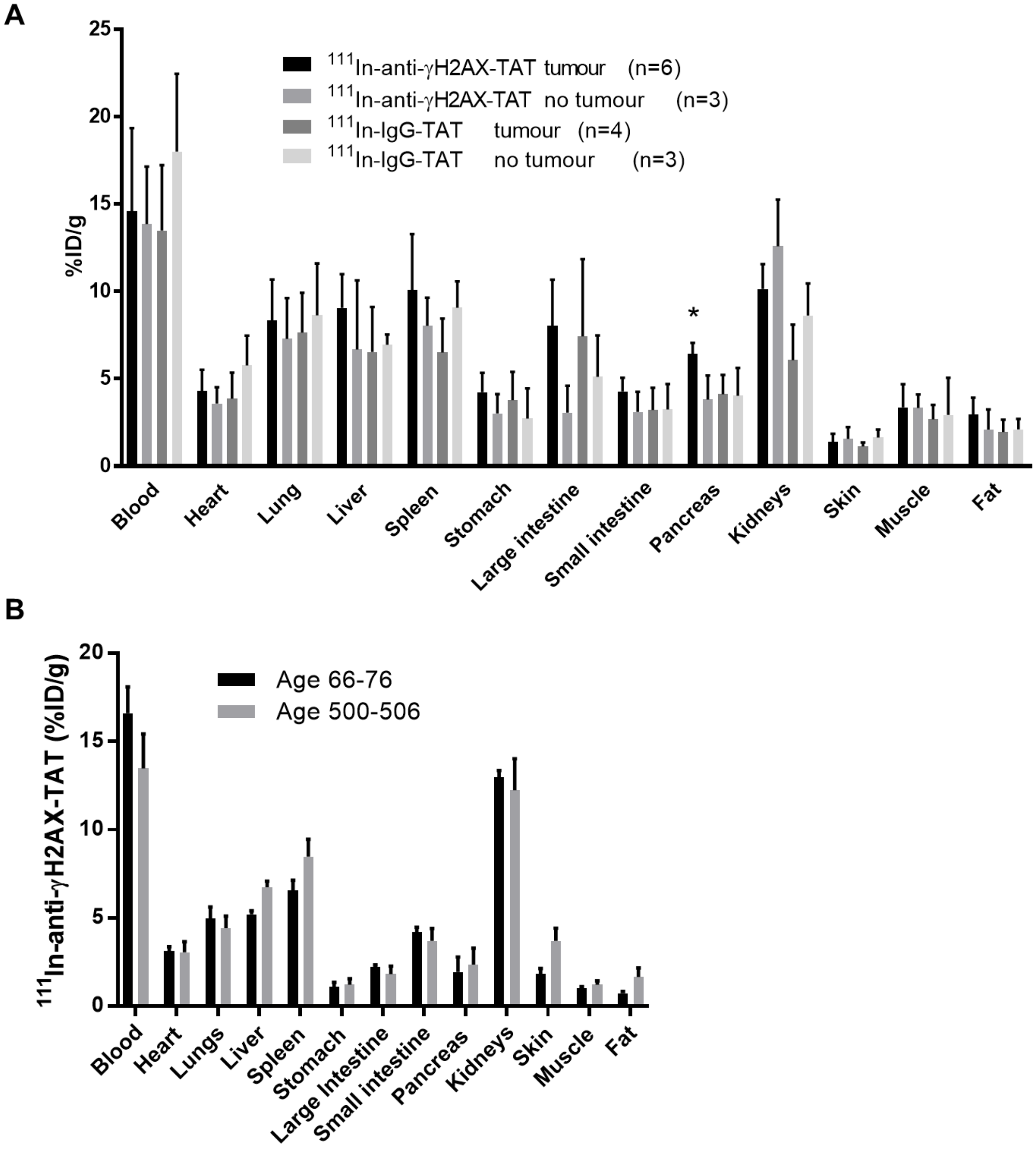

Supplemental Fig. 5 (A) ^{111}In-anti-γH2AX-TAT, but not ^{111}In-IgG-TAT, is taken up more in pancreata of tumour-bearing KPC mice. The pressence of a tumour or lack thereof was confirmed on necropsy. (P = 0.015) (B) The biodistribution of ^{111}In-anti-γH2AX-TAT is not significantly different in older Balb/c wild type animals compared to younger mice.*

Supplemental Fig. 6

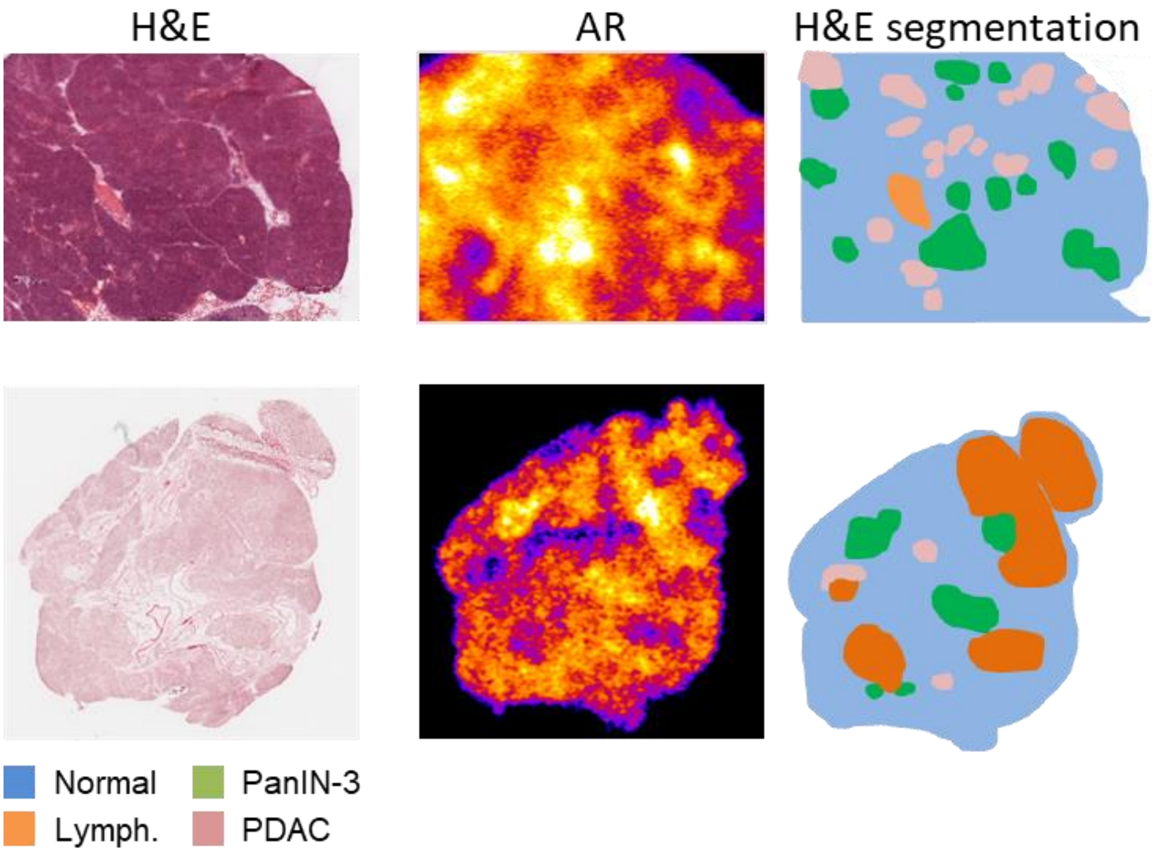

Supplemental Fig. 6. As in Figure 4 A-C. Additional sections.

Supplemental Fig. 7

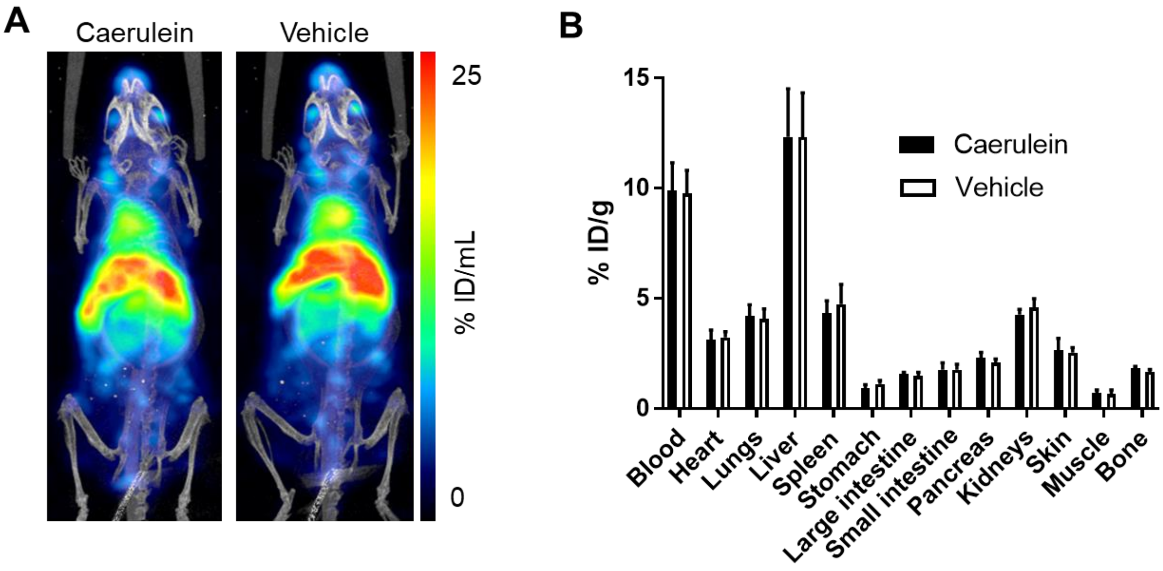

Supplemental Fig. 7 (A) Representative SPECT/CT images of Balb/c mice, treated with caerulein or vehicle control, and imaged 24 h after administration of ^{111}*In-anti-γH2AX-TAT (5 MBq/µg) (B) Ex vivo biodistribution results follwing imaging in A.*

Supplemental Fig. 8

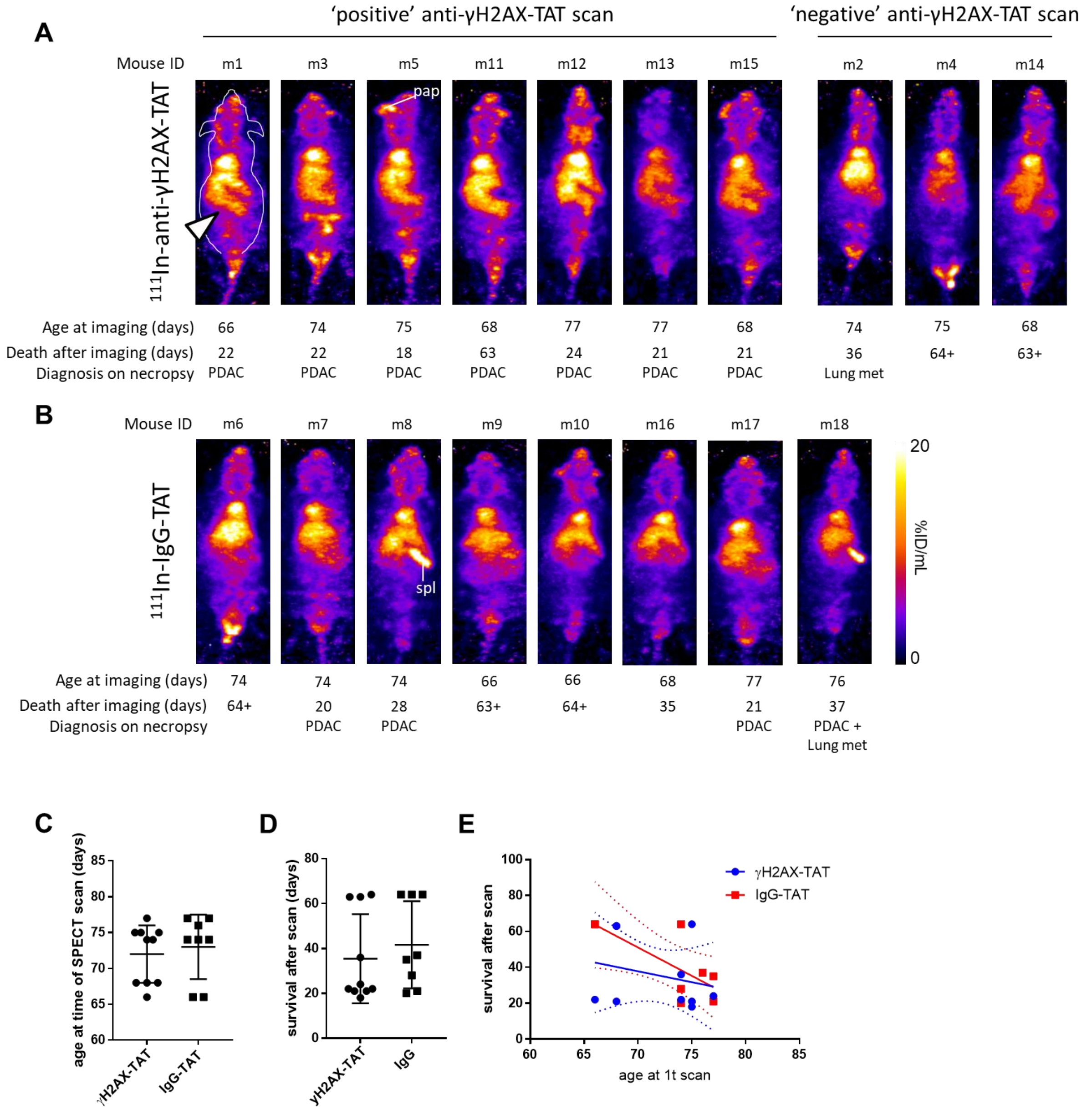

Supplemental Fig. 8 (A) KPC mice aged between 66-77 days were imaged by SPECT, 24 h after intravenous administration of ^{111}In-anti-γH2AX-TAT. Age at the time of imaging, the length of survival before clinical symptom endpoints were reached, and the diagnosis at necropsy are indicated for each mouse (Coronal MIPs are shown, an outline of a mouse is indicated for the first animal only). (B) Similar as (a), but using ^{111}In-IgG-TAT control compound. (C) The age at time of imaging was not significantly different between the different groups (ANOVA; P=0.99). (D) Survival after imaging was not significantly different in animals imaged with ^{111}In-anti-γH2AX-TAT or ^{111}In-IgG-TAT (P=0.51) (E) Survival time after imaging did not correlate with the age at the time of the scan, in this set of animals.
